# Oroxylin A Reduces Vasoconstriction in Rat Aortic Rings through Promoting NO Production and NOS Protein Expression via Estrogen Receptor Signal Pathway

**DOI:** 10.1155/2020/9257950

**Published:** 2020-01-30

**Authors:** Jingtian Qu, Fang Liu, Xuezhu Zhang, Jialong Wang

**Affiliations:** ^1^First Teaching Hospital of Tianjin University of Traditional Chinese Medicine, 314 Anshanxi Road, Nankai District, Tianjin 300193, China; ^2^Guang'anmen Hospital, China Academy of Chinese Medical Sciences, 5 Beixiange, Xicheng District, Beijing 100053, China

## Abstract

Oroxylin A, a flavonoid, is naturally produced in many medicinal plants. Our previous study identified it as a phytoestrogen. Based on this, the present study investigated its vasoconstriction reducing effects and whether the action was mediated by the estrogen receptor (ER) signal pathway. Long-term in vitro treatment with oroxylin A reduced Ach-induced vasorelaxation and NE-mediated or KCl-mediated contractile responses in rat aortic rings. These effects were interfered by an ER inhibitor ICI 182,780. Rat cardiac microvascular endothelial cells (CMECs) and aortic vascular smooth muscle cells (VSMCs) were used to study the possible underlying mechanisms. Oroxylin A activated the ER signal pathway. In CMECs, it increased NO production and eNOS protein expression. In VSMCs, it promoted NO production and iNOS protein expression. These effects were also inhibited by ICI 182,780. Besides, oroxylin A stimulated ER*α* and ER*β* protein expression in CMECs and VSMCs. All these findings suggest that the ER signal pathway takes part in the vasoconstriction reducing effects of oroxylin A.

## 1. Introduction

It has been known for many years that exogenous estrogen exerts protective effects on the vasculature in premenopausal women receiving estrogen replacement therapy [1]. These protections have been found to be associated with its direct effects on blood vessels in part at least [2]. However, sustained exposure to estrogen is a risk factor for blood clots, endometrial cancer, and breast cancer [3, 4]. Therefore, it is important to search for a safe and effective selective regulator of ER for the estrogen replacement therapy to make up for the shortage of estrogen.

Oroxylin A is a flavone naturally produced in many medicinal plants, such as Scutellariae Radix and Oroxylum indicum. Study has found that it had anticancer and cardiovascular protective activity. Wei et al. reported that oroxylin A could inhibit breast cancer cells glycolysis-dependent proliferation [5]. Lu et al. reported that oroxylin A could suppress cell adhesion, invasion, and migration in MDA-MB-231 human breast cancer cells [6]. Ku et al. reported that oroxylin A had antithrombotic activities in vitro and in vivo [7]. Besides, oroxylin A lowered the coronary perfusion pressure in the isolated rat heart and exhibited anti-inflammatory effect in RAW 264.7 cells [8, 9].

Our previous research found that oroxylin A had acute vasodilatory effect. It could relax rat thoracic aortas via endothelial NO pathway [10]. However, little attention had been paid to its chronic effects on blood vessels. The present study was performed to investigate the effects of long-term in vitro treatment with oroxylin A on blood vessels.

In addition, we identified oroxylin A as a phytoestrogen recently. It increased both ER*α* and ER*β* activity [8]. Study has shown that long-term in vitro treatment with estrogen could reduce Ach-induced vasorelaxation and attenuate phenylephrine-induced constriction in rat aortic rings, which is related to its effects activating the ER signal pathway in vascular endothelial and smooth muscle cells [11]. Based on this, the present study investigated the possible mechanisms underlying the vasoconstriction reducing effects of oroxylin A.

## 2. Materials and Methods

### 2.1. Reagents

Fetal bovine serum (FBS) and Dulbecco's modified Eagle's medium (DMEM) were purchased from GIBCO (Grand Island, USA); 17*β*-estradiol (E2) was purchased from National Institute for the Control of Pharmaceutical and Biological Products (Beijing, China); N^G^-nitro-L-arginine methylester (L-NAME), ER antagonist ICI 182,780, norepinephrine (NE), and acetyl chloride (Ach) were purchased from Shanghai Aladdin Biochemical Technology Limited liability company (Shanghai, China); anti-eNOS antibody and anti-iNOS antibody were purchased from MDL (Beijing, China); anti-ER*α* antibody and anti-ER*β* antibody were purchased from Abcam (Cambridge, UK); oroxylin A was purchased from Tianjin Wanxiang Hengyuan Biochemical Technology Limited liability company (Tianjin, China); DMSO was purchased from Macklin (Shanghai, China). DMSO was used as a solvent for oroxylin A, E2, and ICI 182,780. Distilled water was used to dissolve for L-NAME, NE, and Ach.

### 2.2. Animals and Ethics Statement

We used male SD rats in this study. Use of animals for the present study was approved by Tianjin University of Traditional Chinese Medicine Animal Care and Use Committee.

### 2.3. Isolation, Culturing, and Identification of CMECs and VSMCs

CMECs were removed from the hearts of the SD rats at 3 to 4 weeks [12]. The cells were cultured in DMEM containing 10% FBS in humidified atmosphere of 5% CO_2_ and 95% air at 37°C. More than 90% of the cells were identified as endothelial cells by immunostaining with CD31 antibody. VSMCs were prepared from thoracic aorta of 2- to 3-month-old male SD rats via the tissue explants method. The cells were cultured in DMEM containing 10% FBS in humidified atmosphere of 5% CO_2_ and 95% air at 37°C. The cells exhibited the typical “hill and valley” growth pattern. More than 90% of the cells were positive for smooth muscle-specific *α*-actin.

### 2.4. Measurement of Nitrite Concentration

The NO level was measured by nitrate reductase test. CMECs or VSMCs were cultured in 96-well culture plates and exposed to different concentrations of oroxylin A (0.5, 5, 20 *μ*M) for 12 h. Nitrite concentration in culture medium was measured according to the manufacture's protocol (Nanjing Jiancheng Bioengineering institute, Nanjing, China). The optical density was measured at 550 nm.

### 2.5. Measurement of eNOS, iNOS, ER*α*, and ER*β* Protein

The eNOS, iNOS, ER*α*, and ER*β* protein were measured by Western Blot. The membrane was probed with Blocking One at 37°C for 12 hours and then blocked with Blocking Two for 1 hour. Protein bands were detected by ECL. Densitometric analyses were performed using chemiluminescence imaging system. Quantification of eNOS, iNOS, ER*α*, and ER*β* protein expression was normalized to the *β*-actin.

### 2.6. Preparation of the Isolated Aorta and Measurement of Aorta Responses

Male SD rats weighing 250–300 g were sacrificed by decapitation. The thoracic aortas were removed from the rats, cleaned of connective tissue, and cut into 4 mm rings rapidly. Each ring was cultured in 24-well culture plates with different concentrations of oroxylin A in DMEM for 12 h and then suspended in organ bath between two parallel stainless hooks. One hook was fixed and the other was connected to a force transducer. The rings were stretched to a basal tension of 2.0 g gradually. The organ bath was filled with 5 mL of Krebs' solution (composition, mM: KCl 4.7, NaCl 118, CaCl_2_ 1.3, MgSO_4_ 1.2, NaHCO_3_ 25, KH_2_PO_4_ 1.2, D-glucose 10) bubbled with 5% CO_2_ and 95% O_2_ to give a pH of approximately 7.4. The temperature in the organ bath was maintained at 37°C. All rings were contracted with KCl (15, 30, 60 mM) or NE (0.01, 0.1, 1 *μ*M) and relaxed by Ach (0.01, 0.1, 1, 10 *μ*M). Aorta relaxations and contractions were displayed on an oscillograph.

### 2.7. Statistical Analysis

Data were presented as mean ± standard deviation of the mean from *n* number of experiments. The data were analyzed by independent samples *t*-test between two groups. A *p* value of less than 0.05 was considered to be significant.

## 3. Results

### 3.1. Vasoconstriction Reducing Effects of Oroxylin A on Rat Aortic Rings

**Figure 1:**
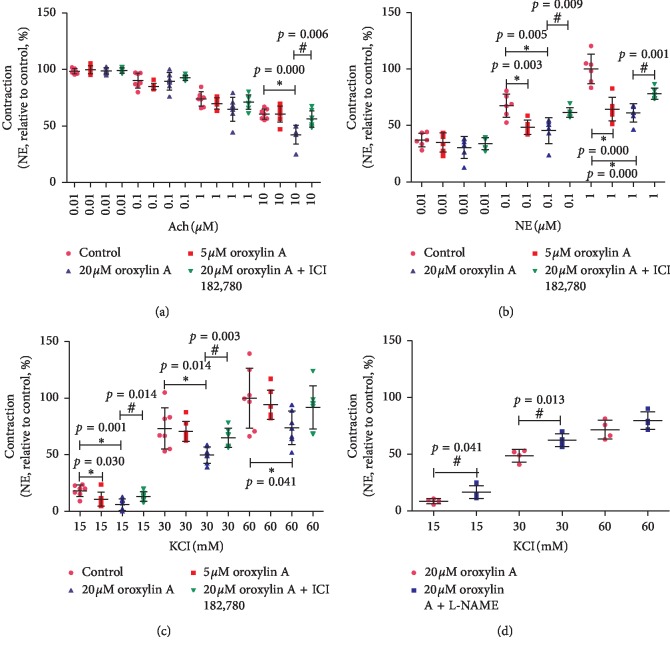
Vasoconstriction reducing effects of oroxylin A on rat aortic rings. (a) Effects of oroxylin A (5, 20 *μ*M, 12 h) on Ach (0.01, 0.1, 1, 10 *μ*M) induced vasorelaxation in the presence or absence of ICI 182,780 (0.1 *μ*M). (b) Effects of oroxylin A (5, 20 *μ*M, 12 h) on NE (0.01, 0.1, 1 *μ*M) induced contractile responses in the presence or absence of ICI 182,780 (0.1 *μ*M). (c) Effects of oroxylin A (5, 20 *μ*M, 12 h) on KCl (15, 30, 60 mM) induced contractile responses in the presence or absence of ICI 182,780 (0.1 *μ*M). (d) Effects of oroxylin A (20 *μ*M, 12 h) on KCl (15, 30, 60 mM) induced contractile responses in the presence or absence of L-NAME (100 *μ*M). Data are shown as mean ± SD. ^*∗*^*p* < 0.05, as compared with the control group. ^#^*p* < 0.05, as compared with the 20 *μ*M oroxylin A group.

To investigate the vasoconstriction reducing effects of oroxylin A, the aortic rings were preincubated with or without oroxylin A (5, 20 *μ*M) for 12 h. Oroxylin A reduced Ach-induced vasorelaxation (*n* = 7, Figure 1(a)) and attenuated NE (0.01, 0.1, 1 *μ*M)-induced (*n* = 6, Figure 1(b)) or KCl (15, 30, 60 mM) induced (*n* = 7, Figure 1(c)) constriction in aorta rings. All these effects were inhibited by ICI 182,780 (0.1 *μ*M, Figure 1(a)*n* = 7, Figure 1(b)*n* = 6, Figure 1(c)*n* = 7). Besides, the effect attenuating KCl-induced constriction of oroxylin A was prevented by a nonselective NO synthase inhibitor L-NAME (100 *μ*M, *n* = 4, Figure 1(d)).

### 3.2. Effects of Oroxylin A on CMEC NO Production, eNOS Protein Expression, ER*α* Protein Expression, and ER*β* Protein Expression

**Figure 2:**
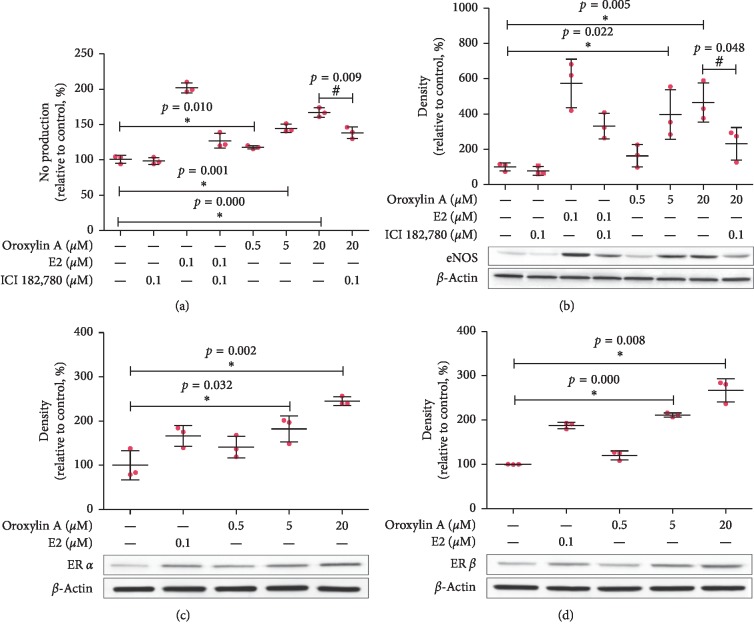
Effects of oroxylin A on CMECs NO production, eNOS protein expression, ER*α* protein expression, and ER*β* protein expression. (a) Effects of oroxylin A (0.5, 5, 20 *μ*M, 12 h) on CMECs NO production in the presence or absence of ICI 182,780 (0.1 *μ*M). (b) Effects of oroxylin A (0.5, 5, 20 *μ*M, 12 h) on CMECs eNOS protein expression in the presence or absence of ICI 182,780 (0.1 *μ*M). (c) Effects of oroxylin A (0.5, 5, 20 *μ*M, 24 h) on CMECs ER*α* protein expression. (d) Effects of oroxylin A (0.5, 5, 20 *μ*M, 24 h) on CMECs ER*β* protein expression. Data are shown as mean ± SD. ^*∗*^*p* < 0.05, as compared with the control group. ^#^*p* < 0.05, as compared with the 20 *μ*M oroxylin A group.

Oroxylin A treatment (0.5, 5, 20 *μ*M) for 12 h increased CMECs NO production and eNOS protein expression, which were prevented by ICI 182,780 (0.1 *μ*M, *n* = 3, Figure 2(a) and Figure 2(b)). Besides, oroxylin A treatment (0.5, 5, 20 *μ*M) for 24 h increased CMEC ER*α* and ER*β* protein expression (*n* = 3, Figure 2(c) and Figure 2(d)).

### 3.3. Effects of Oroxylin A on VSMC NO Production, iNOS Protein Expression, ER*α* Protein Expression, and ER*β* Protein Expression

**Figure 3:**
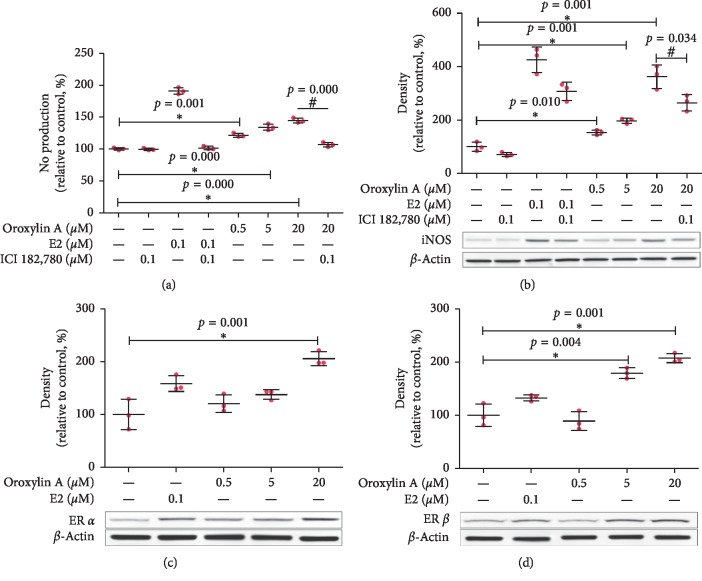
Effects of oroxylin A on VSMCs NO production, iNOS protein expression, ER*α* protein expression, and ER*β* protein expression. (a) Effects of oroxylin A (0.5, 5, 20 *μ*M, 12 h) on VSMCs NO production in the presence or absence of ICI 182,780 (0.1 *μ*M). (b) Effects of oroxylin A (0.5, 5, 20 *μ*M, 12 h) on iNOS protein expression in the presence or absence of ICI 182,780 (0.1 *μ*M). (c) Effects of oroxylin A (0.5, 5, 20 *μ*M, 24 h) on VSMCs ER*α* protein expression. (d) Effects of oroxylin A (0.5, 5, 20 *μ*M, 24 h) on VSMCs ER*β* protein expression. Data are shown as mean ± SD. ^*∗*^*p* < 0.05, as compared with the control group. ^#^*p* < 0.05, as compared with the 20 *μ*M oroxylin A group.

To find out if NO released from VSMCs took part in the constriction inhibiting effect of oroxylin A, we used a nitrate reductase method for VSMCs NO detection. Oroxylin A (0.5, 5, 20 *μ*M) treatment for 12 h increased VSMCs NO production, which were prevented by ICI 182,780 (0.1 *μ*M, *n* = 3, Figure 3(a)). Then, we determined the effect of oroxylin A on VSMCs iNOS protein expression. Oroxylin A (0.5, 5, 20 *μ*M) treatment for 12 h induced VSMCs iNOS protein expression, and the effects were also prevented by ICI 182,780 (0.1 *μ*M, *n* = 3, Figure 3(b)). Besides, oroxylin A treatment (0.5, 5, 20 *μ*M) for 24 h increased VSMCs ER*α* and ER*β* protein expression (*n* = 3, Figures 3(c) and 3(d)).

## 4. Discussion

Most study focused on the acute vasodilatory effect of natural products and little attention had been paid to their chronic effects on blood vessels in vitro. The present study was performed to investigate the chronic effects of oroxylin A on blood vessels. In addition, we identified oroxylin A as a phytoestrogen. It increased both ER*α* and ER*β* activity [8]. Based on this, the present study investigated its vasoconstriction reducing effects and the underlying molecular mechanisms.

Estrogen shows acute effect on blood vessels. It could result in a rapid vasodilatory response through both endothelium-dependent and endothelium-independent mechanisms [13, 14]. Our previous research found that oroxylin A could quickly relax rat thoracic aortas via endothelial NO pathway [10]. However, an ER inhibitor ICI 182,780 (0.1 and 1 *μ*M) could not inhibit this effect. Besides, oroxylin A could not relax rat endothelium-denuded aortic rings precontracted with NE (1 *μ*M) or KCl (60 mM). These findings suggest that ER signal pathway might not be involved in the acute vasodilatory effect of oroxylin A.

Estrogen also shows chronic effects on blood vessels. In endothelium-intact rings, long-term treatment with estrogen could stimulate eNOS protein expression and reduce Ach-induced vasorelaxation. In endothelium-denuded rings, chronic estrogen treatment could promote iNOS protein expression and attenuate phenylephrine-mediated constriction [11]. The present study found that long-term treatment with oroxylin A reduced Ach-induced vasorelaxation and inhibited contractile responses to NE or KCl in rat aortic rings, which were interfered by ICI 182,780. These findings suggest that ER signal pathway was involved in the chronic effects of oroxylin A on rat aortic rings. Long-term treatment with estrogen could stimulate NO production in vascular endothelial and smooth muscle cells. NO plays an important role in the regulation of vasodilation. It could activate soluble guanylyl cyclase catalyzing guanosine triphosphate into cGMP in VSMCs and dilate the blood vessels as a result [15–17]. In this study, we found that chronic treatment with oroxylin A activated the ER signal pathway, leading to increased NO production and NOS protein expression in rat CMECs and VSMCs. Besides, oroxylin A reduced KCl-induced contraction in rat aortas, which were interfered by an ER inhibitor ICI 182,780 and a nonselective NO synthase inhibitor L-NAME. These results suggest that the vasoconstriction reducing effects of oroxylin A might be associated with its action stimulating NO production and NOS protein expression via ER signal pathway in vascular endothelial cells and smooth muscle cells.

The increase in cardiovascular diseases following the onset of menopause has proved that estrogen is a cardiovascular protective agent [18, 19]. However, estrogen replacement therapy was reported to be related to a higher rate of endometrial cancer, breast cancer, and thromboembolic events [20]. Therefore, it is important to search for a safe and effective selective regulator of ER in natural products. Oroxylin A that has anticancer and cardiovascular protective activity might be an alternative in hormone replacement therapy. The present study provided the evidence that oroxylin A could reduce vasoconstriction in rat aortic rings through promoting NO production and NOS protein expression (eNOS protein in endothelial cells and iNOS protein in smooth muscle cells) via ER signal pathway.

However, it should be noted that oroxylin A could induce large resistance arteries vasoconstriction by decreasing circulating NO in endotoxemic rats [21]. Inhibition of LPS-induced iNOS expression in macrophages might play a leading role in this effect of oroxylin A [8]. Besides, oroxylin A shows estrogen-like activity. Although estrogen could promote NO production through increasing iNOS expression in normal VSMCs [22], it could also inhibit iNOS expression and activity in cytokine treated VSMCs [23, 24]. Oroxylin A might have the dual role, so inhibition of cytokine-induced iNOS expression in VSMCs might be the other reason for the decreasing circulating NO effect of oroxylin A. Moreover, endothelial cells are injured with a downregulation of eNOS and an upregulation of iNOS after LPS treatment [25]. The promoting NO production and eNOS protein expression effect of oroxylin A might be damaged and make little contribution to the circulating NO in endotoxemic rats. Oroxylin A might also inhibit iNOS expression in endothelial cells [21]. All these actions may play a role together in the decreasing circulating NO effect of oroxylin A in endotoxemic rats.

## Data Availability

The data used to support our findings are included within the article.

## Supplementary Materials

Supplementary Materials 1. The full blots of eNOS. 2. The full blots of iNOS. 3. The full blots of ER*α* in CMECs. 4. The full blots of ER*β* in CMECs. 5. The full blots of ER*α* in VSMCs. 6. The full blots of ER*β* in VSMCs.
