# Supplementary material for: Oroxylin A Reduces Vasoconstriction in Rat Aortic Rings through Promoting NO Production and NOS Protein Expression via Estrogen Receptor Signal Pathway
Source: Evid Based Complement Alternat Med. 2020 Jan 30;2020:9257950. doi: 10.1155/2020/9257950 (PMC7011478; doi:10.1155/2020/9257950)
Supplement: Supplementary Materials — 1. The full blots of eNOS. 2. The full blots of iNOS. 3. The full blots of ERα in CMECs. 4. The full blots of ERβ in CMECs. 5. The full blots of ERα in VSMCs. 6. The full blots of ERβ in VSMCs. [file 9257950.f1.pdf]

1. The full blots of eNOS

eNOS-1 (140 kDa) (used in the manuscript)

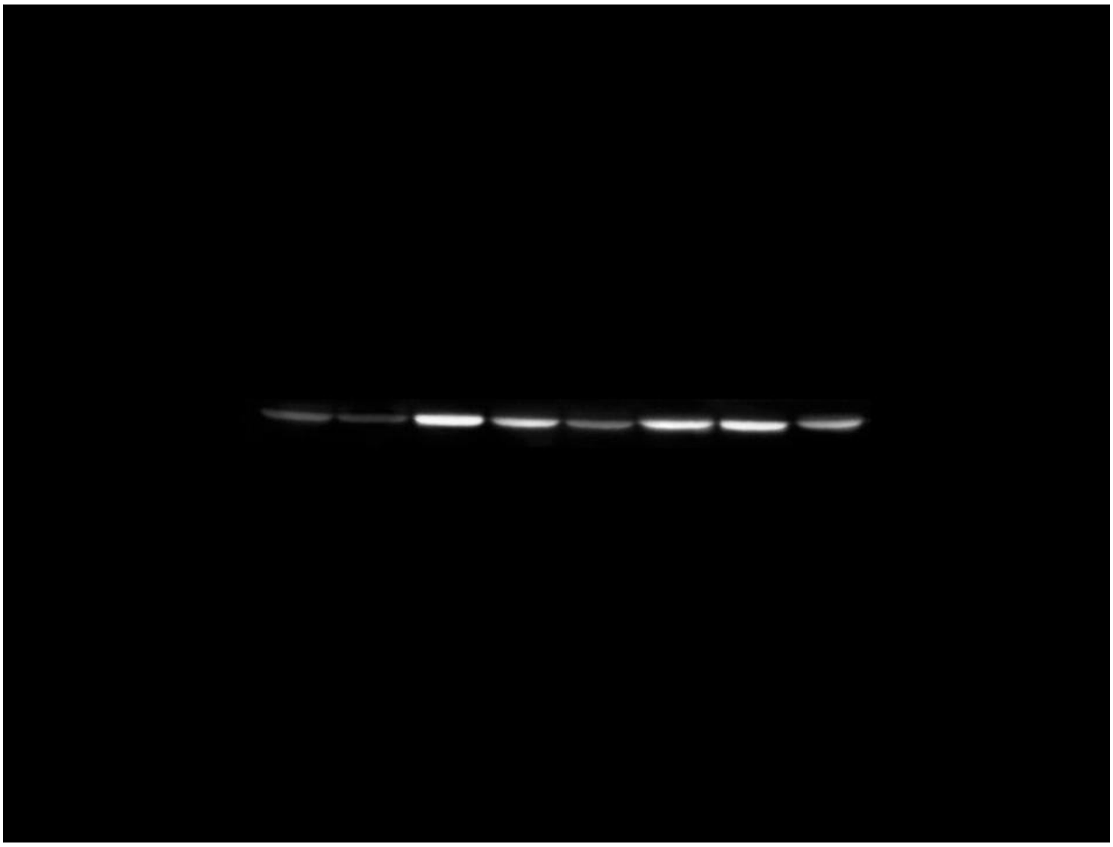

eNOS-2

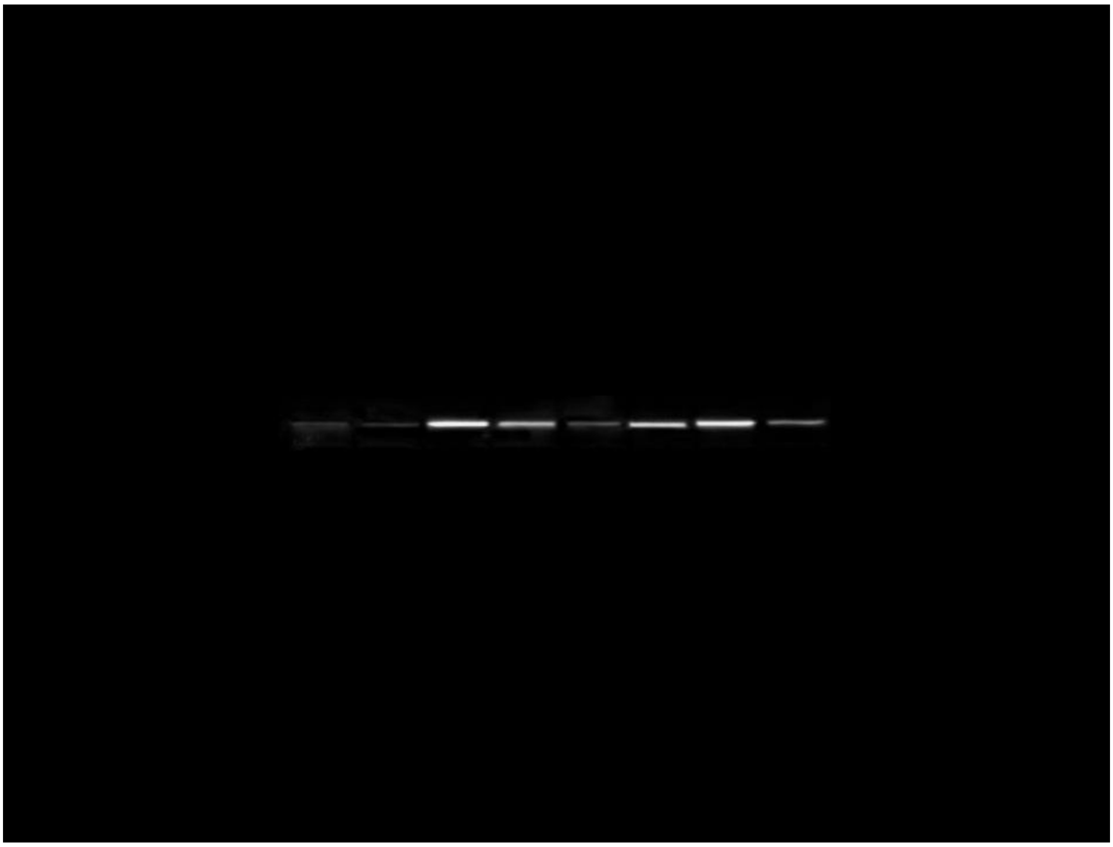

eNOS-3

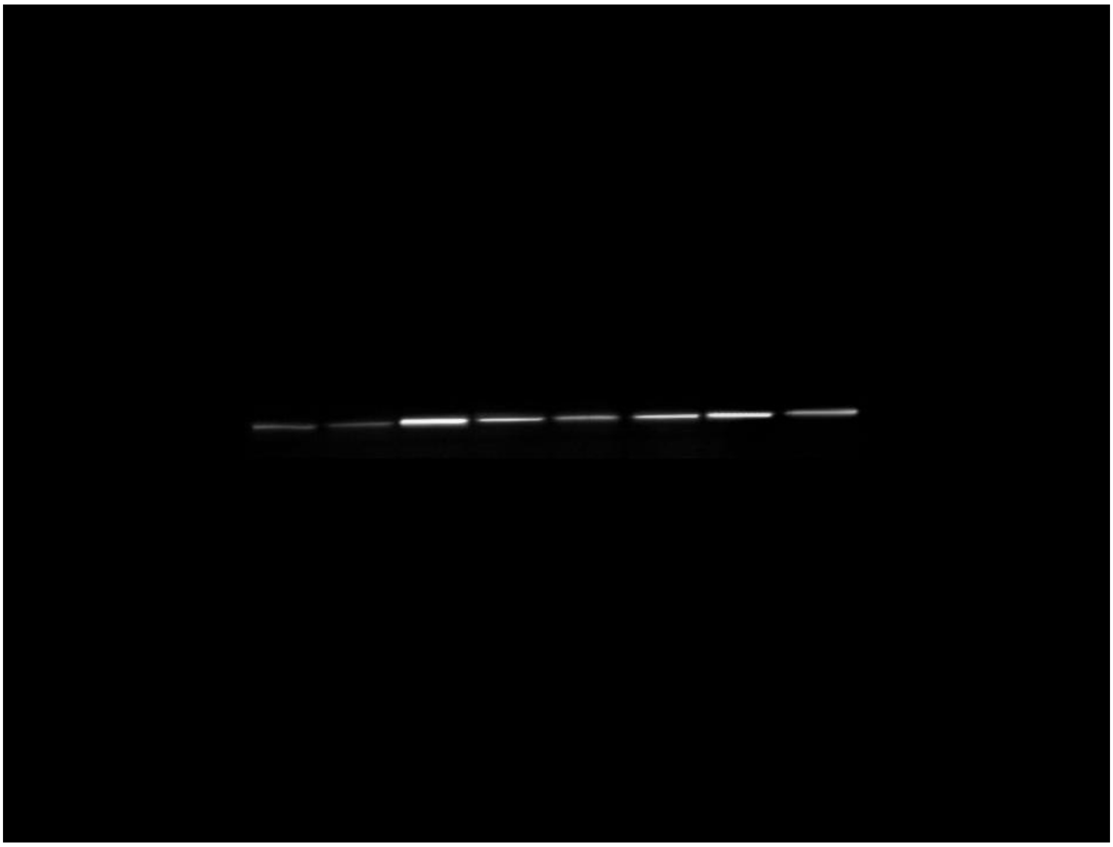

β-actin (42 kDa)（used in the manuscript）

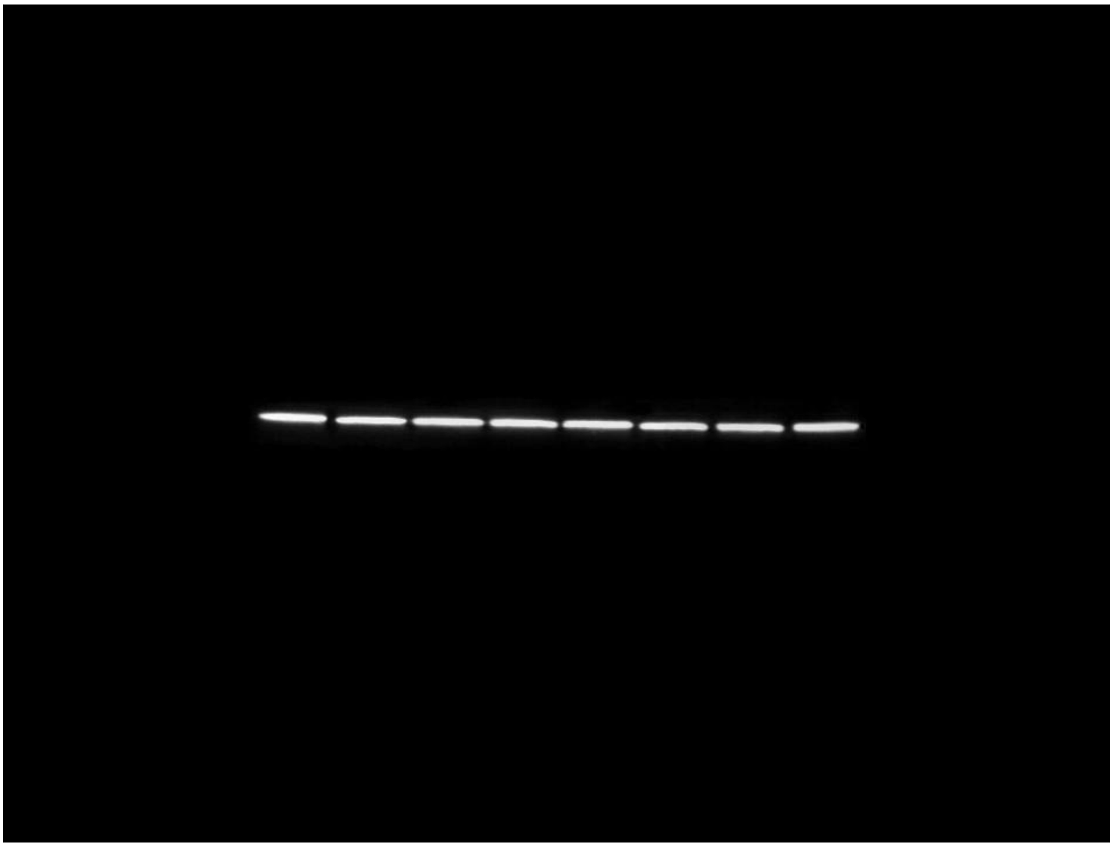

2. The full blots of iNOS

iNOS-1 (140 kDa)（used in the manuscript）

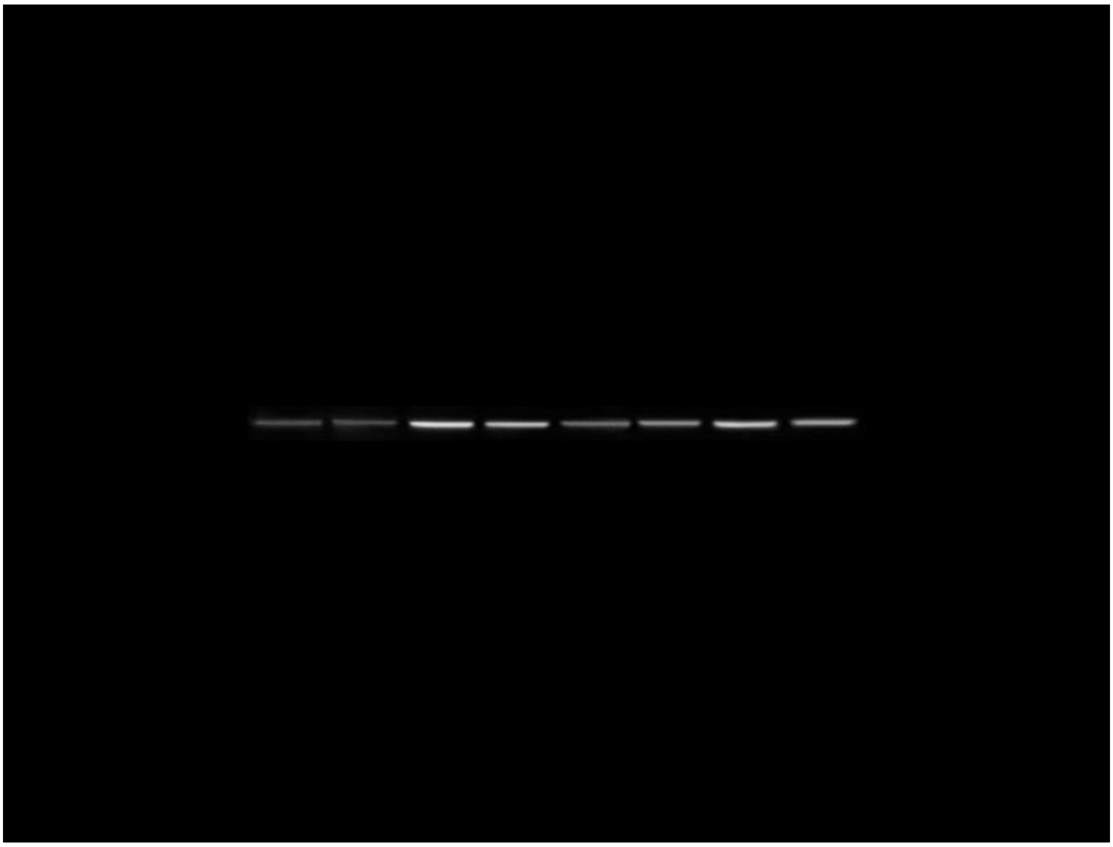

iNOS-2

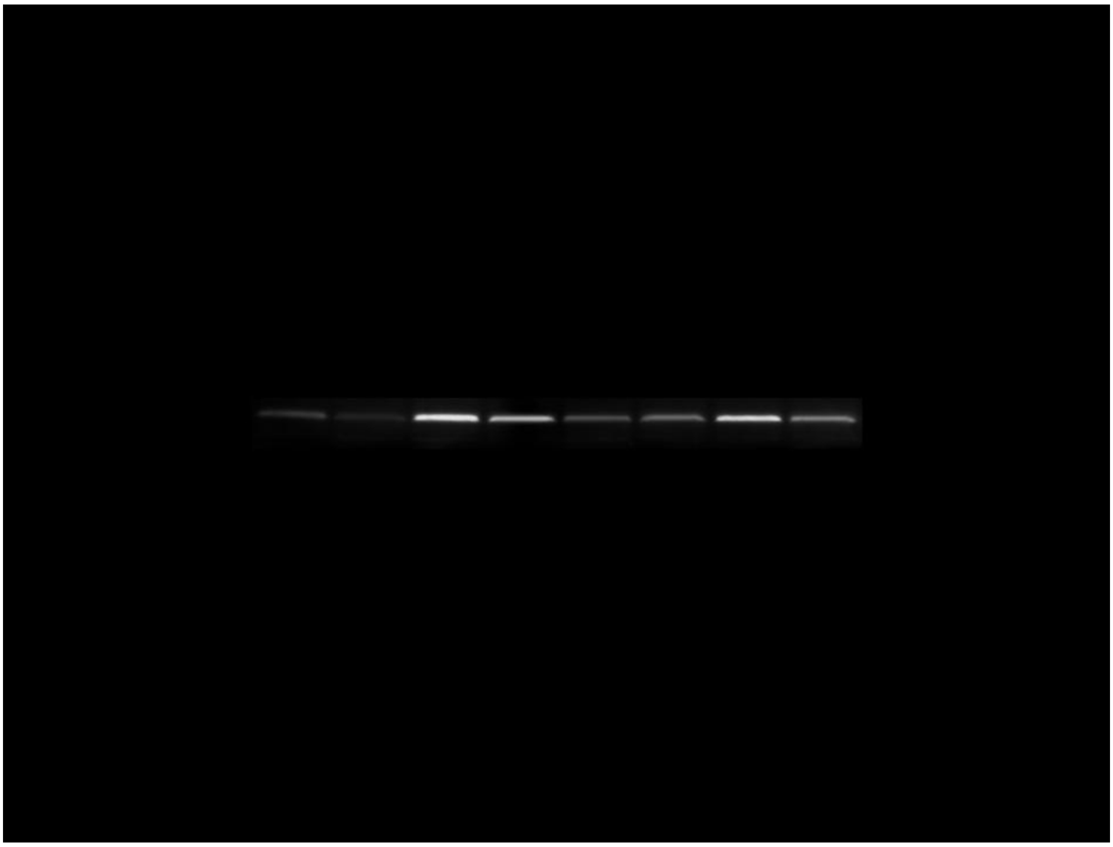

iNOS-3

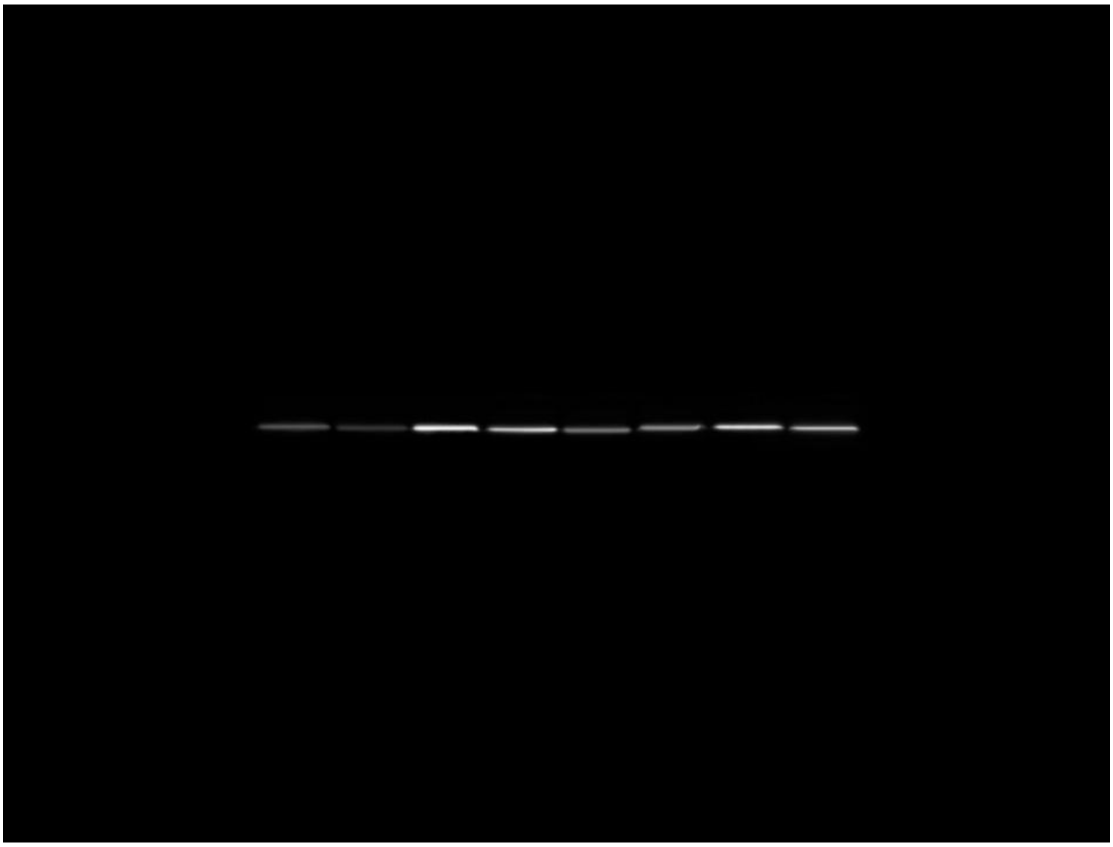

β-actin (42 kDa)（used in the manuscript）

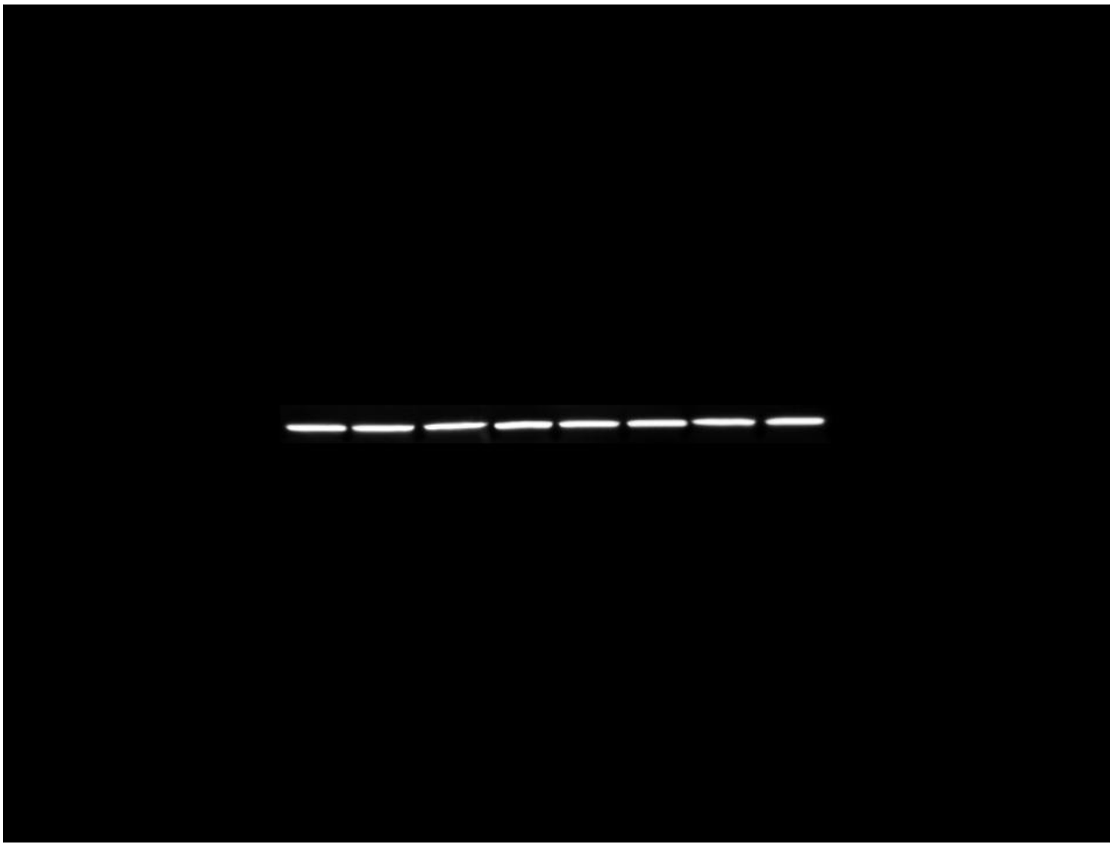

We measured eNOS and iNOS protein one years ago. At that time, we do not know how to develop the marker at the same time developing the target protein. So there is no marker in the full blots of eNOS and iNOS protein.

3.The full blots of ERα in CMECs

ERα-1 (66kDa)

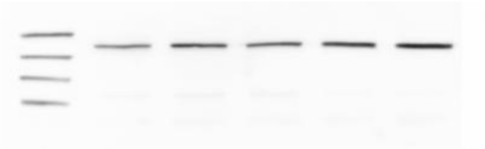

marker from top to bottom is : 70kDa,55kDa,45kDa,35kDa

ERα-2 (66kDa)

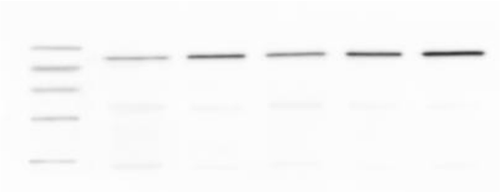

marker from top to bottom is: 70kDa,55kDa,45kDa,35kDa,25kDa

ERα-3 (66kDa) （used in the manuscript）

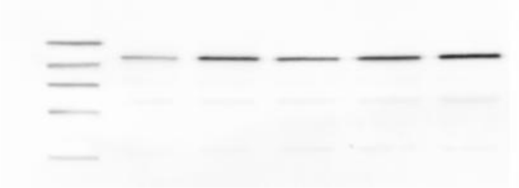

marker from top to bottom is: 70kDa,55kDa,45kDa,35kDa,25kDa

β-actin（42kDa）（used in the manuscript）

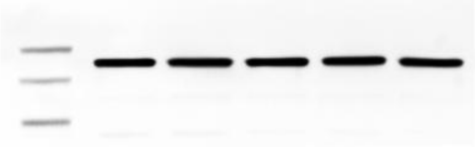

marker from top to bottom is: 45kDa,35kDa,25kDa

4.The full blots of ERβ in CMECs

ERβ-1（48kDa）（used in the manuscript）

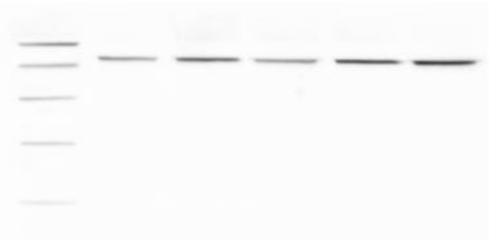

marker from top to bottom is: 55kDa, 45kDa, 35kDa, 25kDa, 15kDa

ER β-2（48kDa）

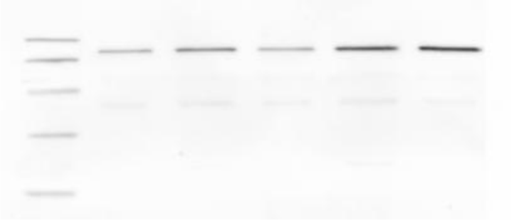

marker from top to bottom is: 55kDa, 45kDa, 35kDa, 25kDa, 15kDa

ER β-3（48kDa）

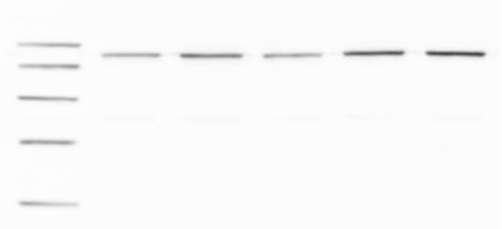

marker from top to bottom is: 55kDa, 45kDa, 35kDa, 25kDa, 15kDa

β-actin（42kDa）（used in the manuscript）

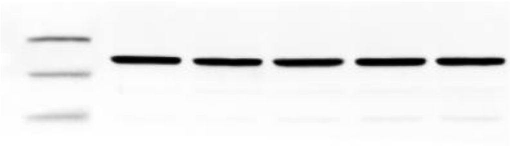

marker from top to bottom is:55kDa, 45kDa, 35kDa

5.The full blots of ERα in VSMCs

ERα-1 (66kDa) （used in the manuscript）

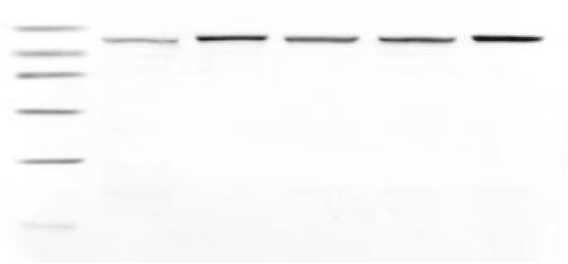

marker from top to bottom is:70kDa, 55kDa, 45kDa, 35kDa, 25kDa, 15kDa

ERα-2 (66kDa)

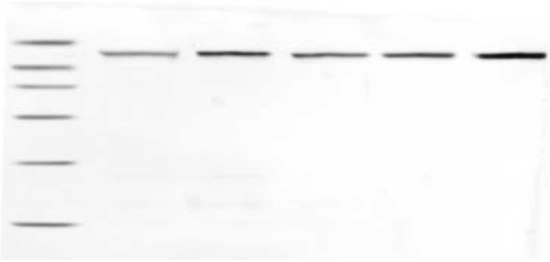

marker from top to bottom is:70kDa, 55kDa, 45kDa, 35kDa, 25kDa, 15kDa

ERα-3 (66kDa)

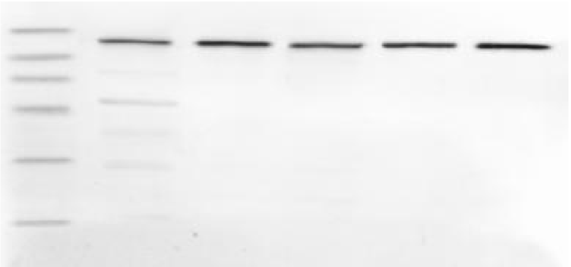

marker from top to bottom is:70kDa, 55kDa, 45kDa, 35kDa, 25kDa, 15kDa

β-actin (42kDa)（used in the manuscript）

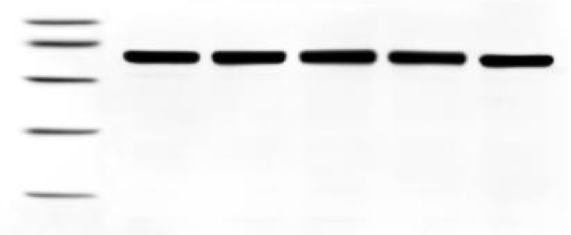

marker from top to bottom is:55kDa, 45kDa, 35kDa, 25kDa, 15kDa

6.The full blots of ERβ in VSMCs

ERβ-1 (48kDa)（used in the manuscript）

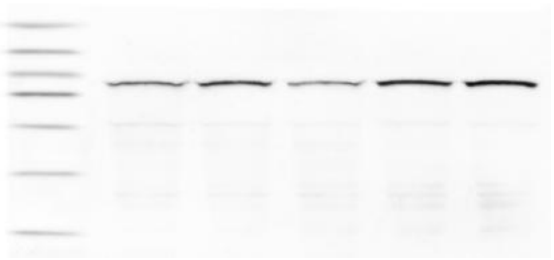

marker from top to bottom is:120kDa, 70kDa, 55kDa, 45kDa, 35kDa, 25kDa, 15kDa

ERβ-2 (48kDa)

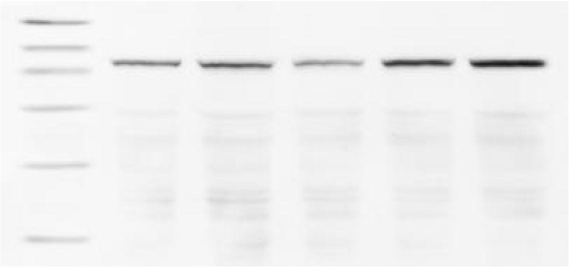

marker from top to bottom is: 70kDa,55kDa,45kDa,35kDa,25kDa,15kDa

ERβ-3 (48kDa)

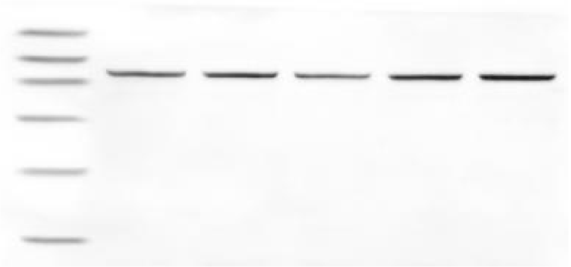

marker from top to bottom is: 70kDa,55kDa,45kDa,35kDa,25kDa,15kDa

β-actin (42kDa)（used in the manuscript）

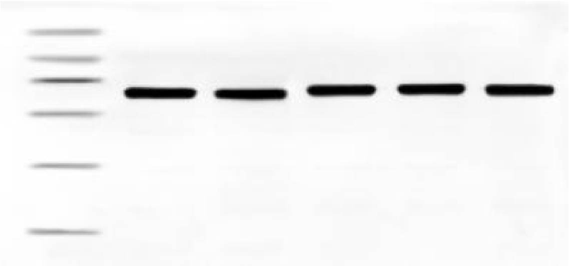

marker from top to bottom is: 70kDa,55kDa,45kDa,35kDa,25kDa,15kDa
